# The Frequency of *Enterobius Vermicularis* Infections in Patients Diagnosed With Acute Appendicitis in Pakistan

**DOI:** 10.5539/gjhs.v7n5p196

**Published:** 2015-02-24

**Authors:** Muhammad Umer Ahmed, Muhammad Bilal, Khurram Anis, Ali Mahmood Khan, Kaneez Fatima, Iqbal Ahmed, Ali Mohammad Khatri, Shafiq-ur-Rehman

**Affiliations:** 1 Ziauddin University and Hospital, Ziauddin Medical College, Karachi, Pakistan; 2 Dow university of health sciences, Karachi, Pakistan; 3 Milton Keynes General Hospital, UK; 4 Department of Sugery Ziauddin University and Hospital, Karachi, Pakistan; 5 Head of Department of Sugery Ziauddin University and Hospital, Karachi, Pakistan

**Keywords:** *Enterobius Vermicularis*, appendicitis, prevalence, Pakistan

## Abstract

**Introduction:**

The main aim of this study was to determine the frequency of *Enterobius Vermicularis* infections and other unique histopathological findings in patients diagnosed with acute appendicitis.

**Materials:**

This retrospective study was conducted in a tertiary care hospital of Karachi, Pakistan over a time period of 9 years from 2005 to 2013. The recorded demographic and histopathological data for the 2956 appendectomies performed during this time frame were extracted using a structured template form. Negative and incidental appendectomies were excluded from the study.

**Results:**

Out of the 2956 patients diagnosed with acute appendicitis, 84 (2.8%) patients had *Enterobius Vermicularis* infections. Malignancy (n=2, 0.1%) and infection with *Ascaris* (n=1, 0.1%) was found very rarely among the patients. Eggs in lumen (n=22, 0.7%), mucinous cystadenoma (n=28, 1.0%), mucocele (n=11, 0.4%), lymphoma (n=9, 0.3%), obstruction in lumen (n=17, 0.6%) and purulent exudate (n=37, 1.3%) were also seldom seen in the histopathological reports.

**Conclusion:**

*Enterobius Vermicularis* manifestation is a rare overall but a leading parasitic cause of appendicitis. Steps such as early diagnosis and regular de worming may help eradicate the need for surgeries.

## 1. Introduction

Considered as one of the surgical emergencies, acute appendicitis normally manifests upon inflammation of the inner lining of the appendix vermiformis, which can spread to other parts of the organ. The suspected cases are frequently treated with appendectomy (Akbulut et al., 2011). The surgical procedure greatly reduces the risk of life-threatening complications such as perforation and sepsis. Epidemiologic studies have revealed that approximately 7 percent of the population will have appendicitis in their life time, with the peak incidence occurring between the ages of 10 and 30 years (Haren, 1999). The causative agents include various physiopathological processes with luminal obstruction considered to be the most important factor. The other less frequent factors include mucinous cystadenoma or mucocele (Demetrashvili et al., 2012), carcinoid tumor (Al & Vajpeyi, 2011), granulomatous diseases (Abdull, 2010), Enterobiasis (Akbulut et al., 2011), Taeniasis (Hafezi & Seifmanesh, 2011), Ascariasis (Sforza et al., 2011), diverticulitis (Manzanares-CampilloMdel, Pardo-García & Martín-Fernández, 2011), adenocarcinoma (O’Donnell, Badger, Beattie, Carson, & Garstin, 2007), lymphoma (O’Donnell, Badger, Beattie, Carson, & Garstin, 2007), and neurogenic appendicopathy (Gupta, Solanki & Vasishta, 2011).

*Enterobius vermicularis* is a cosmopolitan parasite of humans residing in the lumen of the cecum and appendix. As the most common helminthic infection, *Enterobius* Vermicularis accounts for gastrointestinal infections worldwide (Gatti, Lopes & Cevini, 2000). *Enterobius Vermicularis*, also called pinworm, crawls itself to the lumen of the appendix leading to appear some clinical manifestations which resemble acute appendicitis. Obstruction of the narrow appendiceal lumen initiates the clinical illness of acute appendicitis (Green field et al., n.d.). Globally, the reported incidence of *Enterobius* Vermicularis infections in patients with symptoms of appendicitis ranges from 0.2% to 41.8 % (Dahlstrom & Macarthur, 1994). Studies from Nepal (Sah & Bhadani, 2006), Brazil (D. F. Silva, R. J. Silva, M. G. Silva, Sartorelli & Rodrigues, 2007) and Iran (Ramezani & Dehghani, 2007), established the frequency of appendicitis due to Enterobius Vermicularis to be 1.6%, 1.5% and 2.9% respectively.

Having few studies to establish the prevalence of *Enterobius Vermicularis* infections in South-East Asia especially Pakistan, we conducted this study to show the possible role of this highly prevalent human round worm as a cause of acute appendicitis. The main aim of this study was to determine the frequency of *Enterobius Vermicularis* infections and other unique histopathological findings in patients diagnosed with acute appendicitis.

## 2. Materials

In this retrospective study, the electronic records of Ziauddin university Hospital were searched in order to identify all patients who had undergone appendectomy to treat an initial diagnosis of acute appendicitis between March 2005 and January2013. The cases that were diagnosed clinically for appendicitis, with supportive evidences from complete blood count (CBC), C - reactive protein (CRP), ultrasound and computed tomography (CT) scan, were included in the study. Patients who received incidental and negative appendectomies were excluded from the study enrollment. The recorded demographic and histopathological data for the 2956 appendectomies performed during this time period were extracted using a structured template form. The different histopathological variables included were purulent exudate, fecaliths, and infiltration of neutrophils, necrosis of wall, perforation, mucosal micro abscess, eosinophilia, obstruction, granulomatous inflammation, lymphoid hyperplasia, malignancy, mucinous cystadenoma, and mucocele, Ascaris, lymphoma and fibrous obliteration. All the histological slides of appendix specimen had been prepared and studied under different magnification of light microscopy. The histopathological observations of the specimens were interpreted individually. The data were analyzed using Microsoft excel v.2010 for tabulation and percentage calculation.

## 3. Results

A total of 2956 patients underwent appendectomy to treat an initial diagnosis of acute appendicitis. The mean age of the patients was 24.8 years with almost 60% of the sample being females. Out of those 2956 patients, 84(2.8%) had infections with *Enterobius Vermicularis*. Malignancy (n=2, 0.1%) and infection with Ascaris (n=1, 0.1%) was found very rarely among the patients. Eggs in lumen (n=22, 0.7%), mucinous cystadenoma (n=28, 1.0%), mucocele (n=11, 0.4%), lymphoma (n=9, 0.3%), obstruction in lumen (n=17, 0.6%) and purulent exudate (n=37, 1.3%) were also seldom seen in the histopathological reports. However, lymphoid hyperplasia (n=1173, 39.7%), fibrous obliteration (n=306, 10.4%), infiltration of neutrophil (n=713, 24.1%) and perforation in the wall (n=306, 10.4%) were much more common findings. Table 1 shows the frequency of the different histopathological and demographical variables found amongst patients with acute appendicitis.

**Table 1:** The frequency of different histopathological and demographical variables found among the acute appendicitis patients

Variables	Frequency	Percentage
**Age (Years)**	
1-10	199	6.7
11-20	846	28.6
21-30	1211	41.0
31-40	370	12.5
41-50	237	8.0
51-60	36	1.2
61-70	52	1.8
71-80	5	0.2
Enterobius *Vermicularis* infections	84	2.8
Females	1728	58.5
Eggs in lumen	22	0.7
Purulent exudates	37	1.3
Fecaliths	29	1.0
Infiltration of neutrophil	713	24.1
Necrosis of wall	91	3.1
Perforation	306	10.4
Mucosal microabscess	17	0.6
Eosinophilia	85	2.9
Obstruction	17	0.6
Granulomatous inflammation	82	2.8
Lymphoid hyperplasia	1173	39.7
Malignancy	2	0.1
Mucinous cystadenoma	28	1.0
Mucocele	11	0.4
Ascaris	1	0.1
Lymphoma	9	0.3
Fibrous obliteration	306	10.4

The mean age of the patients who were positive for *Enterobius Vermicularis* infections was 24.6 years with a higher proportion of females (n=62, 73.8%). More than half of these patients (n=48, 57.1%) were found to have lymphoid hyperplasia. Necrosis of wall (n=8, 9.5%), granulomatous inflammation (n=3, 3.6%), perforation (n=5, 6%) and mucinous cystadenoma (n=1, 1.2%) were seldom seen. No patient showed signs of malignancy or mucosal micro abscess. Table 2 shows the characteristics of all the acute appendicitis patients who had Enterobius Vermicularis infections.

**Table 2:** Depicts the characteristic of acute appendicitis patients who had Enterobius Vermicularis Infections

Variable	Frequency	Percentage
Age(years)	
1-10	16	19.0
11-20	25	29.6
21-30	20	23.8
31-40	7	8.3
41-50	9	10.7
51-60	3	3.6
61-70	4	4.8
Female	62	73.8
Eggs in lumen	15	17.9
Purulent exudate	8	9.5
Fecaliths	15	17.9
Infiltration of neutrophil	33	39.2
Necrosis of wall	8	9.5
Perforation	5	6.0
Mucosal micro abscess	0	0.0
Eosinophilia	12	14.3
Obstruction	1	1.2
Granulomatous inflammation	3	3.6
Lymphoid hyperplasia	48	57.1
Malignancy	0	0.0
Mucinouscystadenoma	1	1.2
Mucocele	0	0.0
Ascaris	0	0
Lymphoma	0	0
Fibrous obliteration	8	9.6

## 4. Discussion

Vermiform appendix is a vestigial organ having functions of immunological aspects in the abdominal cavity which varies in length and position from person to person. It is often manifested by many disease processes like diverticulitis, carcinoma and appendicitis (Uttam et al., 2009). Gastro intestinal manifestations because of *Enterobius Vermicularis* are classified amongst the most commonlyoccurring problems worldwide (Gatti, Lopes, & Cevini, 2000).*Enterobius vermicularis* infection has been seen to affect people of all ages regardless of the socio economic level; however a strong predilection amongst children has been found (Arca, Gates, Groner, Hammond, & Caniano, 2004). Amongst people with symptoms of excruciating perianal pruritus accompanied with restlessness, loss of appetite and insomnia, *Enterobius Vermicularis* infection must be suspected. Diagnosis may be aided by detection of eggs on stool microscopy, visualization of the parasite directly or by the night time application of cellophane tape in the peri-anal area. Pinworms manifest meticulously in the bowel and according to some retrospective studies it is the most trivial worm residing in the appendix. Leading to a myriad of pathological derangements including inflammation, lymphoid hyperplasia, and subsequently complications like peritonitis and gangrene (Silva et al., 2007) The missing link between pinworm infestation and appendicitis was first illustrated in the year 1899 by Still GF (Still GF) following which there have been many studies highlighting this strong relationship (Marjorie, Robert, Jonathan, Sue, & Donna, 2004; Wiebe, 1991; Sah & Bhadani, 2006).

The reported occurrence of pinworm infestations, in patients with appendicitis ranges from 0.2-41.8% worldwide (20). We found that out of the total 2956 appendectomies performed about 84 patients (2.8%) showed histopathological evidence of appendicitis due to *EnterobiusVermicularis*. A retrospective analysis from Turkey showed that out of the total 190 appendectomies performed, 6 specimens (3.2%) revealed parasites, out of which 4 were due to pinworms (Aydin, 2007). This fact was further reimbursed Yildrim et al (Yildirim, Nursal, Tarim, Kayaselcuk, & Noyan, 2005) and Isik B et al. (2007) who reported a similar percentage (3.8%). In Nepal appendicitis secondary to pinworms was identified in upto 1.6% of the total patients who had undergone appendectomies (Sah & Bhadani, 2006). A 10 year study in Brazil yielded 24 cases out of 1600 (Silva et al., 2007) compared with Iran in which around 3% of the patients had Enterobius Vermicularis infections (Ramezani & Dehghani, 2007). Moreover, a slightly higher percentage of 4.0% was found in the Denmark population (Wiebe, 1991). Therefore it can be eluded that our data is fairly consistent with the previous international studies.

We also found that acute appendicitis due to *Enterobius Vermicularis* was more commonly observed amongst the female and younger population. This observation is supported by Marjorie JA etal (Marjorie, Robert, Jonathan, Sue, & Donna, 2004), who also stated females to be the more vulnerable gender. Moreover, Weibe BM et al (Wiebe, 1991) also reported the age group of 6-15 years to be affected mostly, with a major inclination again towards the female gender. In addition, a study conducted in Australia showed the frequency of appendicitis due *to Enterobius Vermicularis* to be 1.9% in malesas compared with 4.6% in females (Dahlstrom & Macarthur, 1994). Apart from this, the prevalence amongst individuals lessthan 14 years of age have also been expressed as 1.5% by William et al. (William & Dixon, 1988), 0.9% by Agarwala et al. (Agarwala & Liu, 2003) and 1.4% by Arca et al. (Arca, Gates, Groner, Hammond, & Caniano, 2004).

From a histo-pathological aspect, our study showed a relatively high frequency of infiltration of neutrophils, eosinophilia, fecaliths and eggs in the lumen as the microscopic reason for appendicitis amongst specimens having Enterobius Vermicularis infections. Similarly, a study in Iran also established purulent exudates, fecaliths and neutrophil infiltration as the most commonly observed findings (GhMowlavi et al., 2004). Sayavashi (1997) also reported purulent exudate to be the most consistent pathological cause while Yildrim et al concluded that appendicitis due to *Enterobius Vermicularis* is mainly because of luminal obstruction or due to hypersensitivity (Gupta, Solanki, & Vasishta, 2011). On the other hand, Dahlstorm concluded that mucosal infiltration by the worm is not a factor contributing to the disease (Dahlstrom & Macarthur, 1994). In addition, results by Budd show that appendicitis due to *Enterobius Vermicularis* usually manifests as signs of chronic inflammation histo pathologically (Budd & Armstrong, 1987). In a nutshell, it could be stated that exact microscopic mechanism of action of *Enterobius Vermicularis in appendicitis is debated* (Surmont & Liu, 1995).

To the best of our knowledge our research is amongst the few to encompass a large sample size of 2956 cases and amalgamate data of an extensive span of 9 years to report the frequency of *Enterobius Vermicularis* infections along with other unique histopathological findings. However there are limitations to this study which must be considered. Firstly, we did not enumerate and report the exact signs and symptoms with which the patients presented with. Secondly, we were also unable to establish that whether male or female pinworms were obtained in the specimens and also did not keep into account the exact season in which the patients presented with and thereby being unable to show a significant seasonal variability with the disease manifestation. Thirdly, keeping in mind the main aim of this study, we did not do advance statistics to establish an association between different variables with incidence of Enterobius Vermicularis infections in acute appendicitis patients.

In conclusion it can be stated that the occurrence of *Enterobius Vermicularis* infections in appendectomy is a unique finding. In developing countries such as Pakistan, where Helminthic infection is a common problem, measures such as regular de worming and symptomatic awareness programs may help eradicate infection and therefore in the long run may help avoid surgery. Efforts such as early diagnosis and prompt treatment may also lower morbidity and mortality associated with the disease. In addition, further work is also required in particular to the histo pathologic aspect so as to clearly highlight the main mechanism behind the disease. Moreover other helminthic infections contributing towards appendicitis must also be explored. All these measures will ultimately help reduce the disease burden on the community.
